# A first-in-human phase I trial of daily oral zelenirstat, a N-myristoyltransferase inhibitor, in patients with advanced solid tumors and relapsed/refractory B-cell lymphomas

**DOI:** 10.1007/s10637-024-01448-w

**Published:** 2024-06-05

**Authors:** Randeep Sangha, Rahima Jamal, Jennifer Spratlin, John Kuruvilla, Laurie H. Sehn, Erwan Beauchamp, Michael Weickert, Luc G. Berthiaume, John R. Mackey

**Affiliations:** 1 Cross Cancer Institute, Edmonton, AB Canada; 2 Centre Hospitalier de l’Université de Montréal, Centre de Recherche du CHUM, Montreal, QC Canada; 3 Princess Margaret Cancer Centre, Toronto, ON Canada; 4 BC Cancer Centre for Lymphoid Cancer, Vancouver, BC Canada; 5 Pacylex Pharmaceuticals Inc., Edmonton, AB Canada; 6 University of Alberta, Edmonton, AB Canada; 7 Cross Cancer Institute, 11560 University Avenue NW, Edmonton, AB T6G 1Z2 Canada

**Keywords:** N-myristoyltransferase inhibitors, Zelenirstat, PCLX-001, Non-Hodgkin iymphoma, Phase I, First-in-human, Dose escalation, Pharmacokinetics, Colon cancer, Overian cancer, Appendiceal carcinoma

## Abstract

Myristoylation, the N-terminal addition of the fatty acid myristate to proteins, regulates membrane-bound signal transduction pathways important in cancer cell biology. This modification is catalyzed by two N-myristoyltransferases, NMT1 and NMT2. Zelenirstat is a first-in-class potent oral small molecule inhibitor of both NMT1 and NMT2 proteins. Patients with advanced solid tumors and relapsed/refractory (R/R) B-cell lymphomas were enrolled in an open label, phase I dose escalation trial of oral daily zelenirstat, administered in 28-day cycles until progression or unacceptable toxicity. The endpoints were to evaluate dose-limiting toxicities (DLT) to establish a maximum tolerated dose (MTD), pharmacokinetic parameters, and anticancer activity. Twenty-nine patients were enrolled (25 advanced solid tumor; 4 R/R B-cell lymphoma) and 24 were DLT-evaluable. Dosing ranged from 20 mg once daily (OD) to 210 mg OD without DLT, but gastrointestinal DLTS were seen in the 280 mg cohort. MTD and recommended phase 2 dose were 210 mg OD. Common adverse events were predominantly Gr ≤ 2 nausea, vomiting, diarrhea, and fatigue. Plasma concentrations peaked at 2 h with terminal half-lives averaging 10 h. Steady state was achieved by day 15, and higher doses achieved trough concentrations predicted to be therapeutic. Stable disease as best response was seen in eight (28%) patients. Progression-free survival and overall survival were significantly better in patients receiving 210 mg OD compared to those receiving lower doses. Zelenirstat is well-tolerated, achieves plasma exposures expected for efficacy, and shows early signs of anticancer activity. Further clinical development of zelenirstat is warranted.

## Introduction

The two human N-myristoyltransferases (NMT), NMT1 and NMT2, add the fourteen-carbon fatty acid, myristate, to the N-terminal glycine residue of proteins, stabilizing them from degradation and targeting them to intracellular membranes [1]. More than 200 human proteins undergo myristoylation [1]. These include key proteins that regulate cell growth and apoptosis, including Src and seven of the human Src family of protein tyrosine kinases (SFKs; Blk, Fgr, Fyn, Hck, Lck, Lyn, and Yes); if these proteins fail to undergo myristoylation, they are rapidly degraded and inactivated by a glycine-specific N-degron [2, 3]. Aberrant myristoylation has been identified in many common cancers [4–6], and inhibitors of NMT have been proposed as anti-cancer therapeutics [4].

In vitro preclinical studies using zelenirstat (previously known as PCLX-001 and DDD86481), a potent small molecule inhibitor of both human NMT proteins, induced dose and time-dependent apoptosis in cancer cells at concentrations lower than those required to kill normal cells. Zelenirstat was cytotoxic or cytostatic across a wide variety of cultured human cancer cells, with high sensitivity noted in a broad array of hematologic malignancies including B-cell non-Hodgkin lymphoma (NHL) and acute myelogenous leukemia (AML) [5]. Comparative transcriptomic analysis of 1200 zelenirstat sensitive and insensitive cell lines identified a myristoylation inhibition sensitivity signature that predicted those cancers most likely to respond to NMT inhibitor therapy [7, 8]. The cancer with the highest sensitivity scores included diffuse large B-cell lymphoma (DLBCL), AML, testicular germ cell tumors, squamous cell carcinoma of the lung, colorectal carcinomas, basal subtype breast cancers, and ovarian carcinoma.

In mice-bearing established human tumor xenografts, zelenirstat achieved tumor growth inhibition in cell line derived xenografts from breast [6] and lung carcinomas (Pacylex Inc., data on file). However, profound tumor regression and complete remissions were seen in patient derived xenograft models of refractory DLBCL, where loss of Src-Lyn and human germinal center-associated lymphoma (HGAL) proteins, which require myristylation to function, inhibited B-cell receptor survival signaling [5]. Zelenirstat produced similarly profound remissions in cell line and patient derived xenograft models of AML. In murine tail-vein injection models of human AML, zelenirstat reduced the leukemic cell burden and dramatically reduced the human leukemic stem cell population [9].

Formal toxicologic evaluation in rats and dogs showed acute gastrointestinal and hematologic toxicities at the highest doses examined. With daily oral dosing at or above the maximally tolerated dose, diarrhea, dehydration, and weight loss were rapidly reversible with discontinuation of zelenirstat and did not recur when re-introduced at lower doses [8]. No cumulative toxicities were identified.

The novel target and mechanism of action, promising pre-clinical findings, and the potential for a convenient oral therapy prompted the advancement of zelenirstat into clinical testing. The primary objectives of this phase I, first-in-human, trial of zelenirstat were to determine (i) safety and tolerability, (ii) the maximum tolerated dose (MTD) and recommended phase II dose (RP2D), and (iii) pharmacokinetic characteristics of daily oral zelenirstat in patients with refractory advanced solid tumors and relapsed/refractory (R/R) B-cell NHL. The secondary objective was to evaluate zelenirstat anticancer activity.

## Methods

### Patient eligibility

Eligible patients were ≥ 18 years old and had (i) advanced solid tumors with progression after at least one prior therapy and who were not eligible for therapies expected to provide clinical benefit, or (ii) B-cell lymphomas including DLBCL, high grade B-cell lymphoma (HGBL), follicular lymphoma (FL) (grades 1 to 3b), mantle cell lymphoma (MCL), and Burkitt lymphoma who had failed at least two prior therapies and were not eligible for therapies expected to provide clinical benefit, such as chimeric antigen receptor (CAR) T-cell therapy or stem cell transplantation. Patients required evaluable or measurable disease, an Eastern Cooperative Oncology Group (ECOG) performance status of ≤ 1, and adequate bone marrow, hepatic, renal, and cardiac function. All other anticancer systemic therapies were required to be discontinued for at least three weeks prior to registration, and patients were expected to be off corticosteroids above prednisone equivalents of 10 mg/day. Strong CYP3A4 inhibitors and inducers were prohibited.

### Study design

This phase I, multicenter, nonrandomized, open-label study of zelenirstat is registered at clinicaltrials.gov as NCT04836195. The study was conducted according to the guidelines of the Declaration of Helsinki, approved by Health Canada and the relevant institutional review boards in each of the four study centres. Written informed consent was obtained from all study subjects. The study was sponsored by Pacylex Pharmaceuticals Inc.

The study is designed in two parts, a dose-escalation phase I, reported here, and two phase IIA dose expansion cohorts, now ongoing in patients with R/R B-cell NHL, and refractory colorectal cancer.

A standard 3 + 3 design was used to assess for MTD and establish RP2D according to a pre-determined dose-escalation schema **(**Table 1**)**. There was no intra-patient dose escalation. The MTD was defined as the dose level below the cohort in which 2 or more patients experienced a first cycle, zelenirstat attributable, dose-limiting toxicity (DLT), and the MTD required confirmation in at least 6 patients.

**Table 1:** Dose-escalation treatment schema

Dose level	Zelenirstat (mg daily)
1	20
2	40
3	70
4	100
5	140
6*	210
7	280

* Three of six DLT evaluable patients initially dosed at 200 mg daily for dose level 6. To reduce the number of capsules for ease of administration, the cohort was amended to 210 mg daily

DLT was defined as any of the following: Grade (Gr) 4 thrombocytopenia, Gr 3 thrombocytopenia with bleeding, Gr 4 neutropenia ≥ 7 days, febrile neutropenia, clinically significant *≥* Gr 3 non-hematologic toxicity, or any significant toxicity warranting a withhold of zelenirstat. Patients who did not receive zelenirstat for at least 21 days during the first cycle for reasons other than DLT were deemed not evaluable and replaced. Toxicity was graded according to the National Cancer Institute Common Toxicity Criteria for Adverse Events (NCI-CTCAE) version 5.0.

### Study drug properties and administration

Zelenirstat is a potent, small molecule inhibitor of human NMT1 and NMT2 proteins. In preclinical models, it has high oral bioavailability, is highly bound to plasma protein, and is metabolized in the liver primarily by CYP3A4 to water soluble metabolites excreted by the kidney (Pacylex, data on file). Zelenirstat has no relevant off-target kinase inhibition [10]. In GLP non-clinical safety testing, diarrhea was dose limiting in multiple daily oral administration, although hematologic toxicity was also seen at the highest exposures [10].

For dose escalation, patients received daily oral zelenirstat on 28-day cycles. It was administered as 10 mg and 70 mg capsules, with instructions to take on an empty stomach each morning with 250 ml of tap water. Patients received zelenirstat until progressive disease, unacceptable toxicity, or withdrawal of consent.

### Study assessments

Baseline imaging and hematology and blood chemistry were obtained within 4 weeks and 1 week of study entry, respectively. Baseline screening included a complete medical history, physical examination, assessment of ECOG performance status, and body weight. CT imaging was used for advanced solid tumors and B-cell NHL; PET-CT was optional for B-cell NHL. Weekly complete blood counts with white blood cell differentiation and blood chemistries were drawn for the first cycle, biweekly for cycle 2, and then prior to each 28-day cycle. Adverse events were evaluated weekly in the first cycle, biweekly for cycle 2, and before each subsequent cycle using the NCI-CTCAE version 5.0.

Tumor response assessments using CT imaging were performed every two cycles. RECIST 1.1 [11] or the Lugano Response Criteria for Lymphoma [12] was used to determine tumor response and disease progression. For B-cell NHL patients with PET-CT at baseline, PET-CT was repeated after the sixth cycle to confirm complete response or disease progression. Patient archival specimens and blood samples were submitted for exploratory molecular analyses.

### Pharmacokinetic sampling and analyses

Peripheral blood samples were collected over six time points in the first 8 h of Cycle 1, Day 1 (C1D1), and again on C1D15. Pre-treatment Day 1 levels were obtained with every subsequent cycle. Zelenirstat plasma concentrations were quantified using a validated ultra-performance liquid chromatography with tandem mass spectrometry detection and analyzed using a nonlinear mixed-effects model.

### Statistical methods

Descriptive statistics were used for demographic, safety, and efficacy data. Categorical data were summarized using frequency counts and percentages. Time-to-event variables were analyzed by Kaplan-Meier methods. Weighted trajectory analysis for health outcomes used the Chauhan methodology [13].

## Results

Between September 2021 through January 2024, 29 patients received a dose of zelenirstat in the dose-escalation cohorts. Baseline demographics and disease characteristics are outlined in Table 2.

**Table 2:** Phase I baseline patient and disease characteristics of patients treated with zelenirstat^*^

Characteristic	No. of patients (*n* = 29)	%
Age, years		
Median	65.0	
Range	37–86	
Sex		
Female	17	58.6
Male	12	41.4
ECOG performance status		
0	16	55.2
1	13	44.8
Previous Systemic Therapies		
Chemotherapy	34	
Immunotherapy	18	
Targeted Therapy	8	
Hormonal Therapy	2	
Prior Systemic Regimens		
Median	4	
Range	1–8	
Tumor Type		
Colorectal adenocarcinoma	8	27.6
B-cell NHL^**^	4	13.8
Ovarian carcinoma^***^	3	10.3
Melanoma	2	6.9
Pancreatic adenocarcinoma	2	6.9
Lung adenocarcinoma	2	6.9
Leiomyosarcoma	1	3.4
Breast adenocarcinoma	1	3.4
Pleural mesothelioma	1	3.4
Anal squamous carcinoma	1	3.4
Prostate small cell carcinoma	1	3.4
Bladder adenocarcinoma	1	3.4
Appendiceal carcinoma	1	3.4
Gallbladder adenocarcinoma	1	3.4

* Zelenirstat 20 mg − 280 mg oral daily on a 28-day cycle

** Three patients with DLBCL and one patient with follicular lymphoma, grade 3b

*** One patient each with clear cell, serous, and NOS histology

*Abbreviations* ECOG, Eastern Cooperative Oncology Group; DLBCL, diffuse large B-cell lymphoma

### Safety, MTD, and RP2D

Twenty-nine patients, who received at least one dose of zelenirstat, were evaluable for safety endpoints. The number of cycles completed ranged from 1 to > 14, with 1 subject continuing on treatment (210 mg daily) at the time of analysis. Medication compliance was high, with patient diaries documenting 90% or higher median compliance across all dose cohorts apart from the 280 mg cohort.

Across dose cohorts up to and including 210 mg, the most common treatment-related adverse events (TRAEs) were gastrointestinal disorders, none of which were dose limiting. Gr 1 and 2 decreased appetite was reported in 11 of the 24 patients (46%), Gr 1 and 2 nausea and diarrhea were reported in 10 subjects (42%), and Gr 1 and 2 vomiting in 9 subjects (37%). Three patients (12%) reported reflux symptoms and abdominal pain. All gastrointestinal toxicities were self-limiting or responsive to routine supportive measures and zelenirstat continued at full dose in each case. All grade fatigue was reported in 11 patients (46%). Gr 2 thrombocytopenia was seen in three patients (12%) but resolved despite continuation of the drug at full dose. No neuropathy was reported. At doses up to and including 210 mg, each of the treatment-related serious adverse events (SAEs) were attributed to progressive disease rather than drug toxicity, including one patient with pleural and pericardial mesothelioma with extensive and progressive mediastinal involvement who experienced a fatal cardiac arrest.

In the 280 mg cohort, five patients began treatment, but two discontinued therapy due to rapid clinical deterioration associated with progression of metastatic mucosal melanoma and prostatic small cell carcinoma. These patients were replaced. Three evaluable patients treated at 280 mg daily experienced first cycle dose-limiting gastrointestinal toxicities, including Gr 3 diarrhea in the setting of biliary stent dysfunction and cholangitis, Grade 3 diverticulitis and abdominal pain in a patient with known diverticular disease, and Grade 3 dehydration. Given these first cycle DLTs, and the absence of first cycle (or any cycle) DLTs seen in the six evaluable patients treated in the 210 mg per day cohort, we established 210 mg as the MTD and RP2D of oral daily zelenirstat.

The distribution of Gr ≥ 3 TRAEs as a function of dose are listed in Table 3. Pre- and post-dose ECG assessments showed no evidence of drug-induced QT prolongation and no trend for increasing QT duration with increasing drug dose (data not shown).

**Table 3:** Grade ≥ 3 treatment-related adverse events for patients treated with at least one dose of zelenirstat

Adverse event^*^	20 mg (*N* = 3)	40 mg (*N* = 3)	70 mg (*N* = 3)	100 mg (*N* = 5)	140 mg (*N* = 3)	210 mg (*N* = 7)	280 mg (*N* = 5)
No. of pts with Gr ≥ 3 TRAEs** (n, %)	0	0	1 (33)	0	0	0	4 (80)
Blood and lymphatic system disorders	0	0	0	0	0	0	1(20)
- Anemia	0	0	0	0	0	0	1(20)
Gastrointestinal disorders	0	0	0	0	0	0	4 (80)
- Abdominal pain	0	0	0	0	0	0	1 (20)
- Appetite loss	0	0	0	0	0	0	1 (20)
- Diarrhea	0	0	0	0	0	0	1 (20)
- Dehydration	0	0	0	0	0	0	1 (20)
- Diverticulitis	0	0	0	0	0	0	1 (20)
- Nausea	0	0	0	0	0	0	1 (20)
- Gastroduodenitis	0	0	0	0	0	0	1 (20)
General disorders	0	0	1 (33)	0	0	0	1 (20)
- Fatigue	0	0	1 (33)	0	0	0	1 (20)
- Fever	0	0	0	0	0	0	1 (20)
Hepatobiliary disorders	0	0	0	0	0	0	1 (20)
- Cholangitis	0	0	0	0	0	0	1 (20)
Metabolism and nutrition disorders	0	0	0	0	0	0	1 (20)
- Hypophosphatemia	0	0	0	0	0	0	1 (20)

^*^ Adverse events at least possibly related to study drug

^**^ Multiple occurrences of an AE in any one pt were recorded once by the highest NCIC-CTCAE grade

### Pharmacokinetic analysis

Twenty-one treated patients were included in the pharmacokinetic (PK) analysis. All patients had measurable plasma zelenirstat concentrations, with the PK parameters listed in Table 4.

**Table 4:** Summary statistics of plasma pharmacokinetic parameters

Dose	Timepoint	No. of evaluable pts	T_max_ (h) (median, range)	C_max_ (ng/ml) (mean, SD)	AUC_0 − 24_ (h*ng/mL) (mean, SD)	t_1/2_ (h) (mean, SD)
20 mg	C1D1	3	2.0 (0.9-4.0)	276 (117)	1583 (231)	6.7 (1.7)
	C1D15	3	1 (0.5–2.1)	359 (118)	2066 (387)	7.4 (2.0)
40 mg	C1D1	3	0.9 (0.5–3.9)	848 (831)	4819 (4997)	9.2 (2.2)
	C1D15	3	2.2 (2.0-4.1)	774 (720)	7060 (6711)	9.7 (3.0)
70 mg	C1D1	3	3.9 (1.0-7.7)	665 (675)	5654 (5232)	9.5 (NC)
	C1D15	3	4.1 (4.0-7.9)	467 (257)	6613 (3526)	NC
100 mg	C1D1	5	2.0 (2.0-3.8)	2188 (1037)	18,936 (9188)	8.0 (2.3)
	C1D15	3	4.0 (2.0–4.0)	2300 (1664)	24,714 (14,020)	12.0 (NC)
140 mg	C1D1	3	1.0 (0.5–1.1)	1769 (1208)	11,883 (8823)	7.2 (0.9)
	C1D15	3	2.0 (1.0–2.0)	1335 (964)	8150 (2846)	10.4 (5.5)
210 mg	C1D1	4	3.0 (1.9-4.0)	1767 (757)	14,654 (6715)	7.8 (2.4)
	C1D15	4	2.0 (1.0-3.9)	2935 (1047)	27,558 (13,349)	7.7 (3.4)
280 mg	Not available					

*Abbreviations* AUC0-24, area under the plasma concentration-time curve; Cmax, maximum concentration; T_1/2_, half-life; Tmax, time to maximum serum concentration; PK = pharmacokinetic; SD = standard deviation; NC = not calculated; C, cycle 1; D, day

Following single oral administration of 20 mg to 210 mg zelenirstat on C1D1, plasma concentrations of zelenirstat steadily increased reaching peak levels with median T_max_ values ranging from 1 to 4 h across the dose cohorts. After repeated daily dosing, zelenirstat T_max_ values on C1D15 were comparable to values obtained on C1D1. The median T_max_ range was slightly wider (1 to 6 h) in patients taking proton pump inhibitors (PPI) with a maximum T_max_ value of approximately 8 h. After reaching peak concentrations (C_max_) on C1D1, zelenirstat was eliminated from plasma with mean terminal half-life ranging from 6.7 to 9.5 h across the dose cohorts. Zelenirstat half-life values on C1D15 (7.67 to 12.0 h) were comparable to values obtained on C1D1. One patient in the 40 mg cohort had prior pancreatico-duodenal resection and had limited oral absorption of zelenirstat but was included in these analyses. Plasma concentrations (C_max_ and AUC values) tended to be lower in patients on PPIs compared to patients not taking PPIs. The lower exposure in patients on PPIs is likely due to reduced absorption of zelenirstat as half-life was roughly similar in patients on PPIs and those who were not taking PPIs.

Systemic exposure to zelenirstat (C_max_ and AUC values) tended to increase with increasing dose over the dose range of 20 mg to 210 mg. Dose-proportionality over this dose range was not supported by statistical analysis due to the limited number of subjects and the variability introduced by the concomitant use of PPIs. The majority of patients had limited accumulation of zelenirstat (< 2-fold) following repeated administration for 14-days, with accumulation ratios for C_max_ (Ar_Cmax_) and AUC_0 − 24_ (Ar_AUC0−24_) values ranging from 0.41 to 1.72 and 1.0 to 1.9, respectively. Three subjects had accumulation ratios for both (Ar_Cmax_ and Ar_AUC0−24_) between 3- and 6-fold. Based upon pre-dose concentrations over multiple cycles, no accumulation was apparent beyond Day 15. Time to steady state was assessed by visual observation of pre-dose zelenirstat concentrations. Steady state, with some variability, was achieved by day 8 to 15 of dose administration in most patients.

Although zelenirstat is metabolized by the liver to water soluble metabolites which are primarily excreted primarily by the kidney, there were no apparent relationships noted between PK and hepatic or renal function tests. The relationships among pharmacokinetic parameters that reflect zelenirstat exposure and clinical toxicities were explored, but none were found (data not shown).

### Anticancer activity

Ten treated patients lacked response assessment imaging due to rapid clinical progression and/or functional deterioration (one in the 20 mg cohort, one in the 70 mg cohort, two in the 100 mg cohort, and two in the 210 mg cohort, and 4 in the 280 mg cohort). Stable disease (SD) as best response was seen in eight patients (28%), Table 5. SD was noted in a single patient treated at 40 mg until week 16, at 140 mg until week 24, and at 280 mg until week 16. Two patients with refractory ovarian cancer treated at 210 mg achieved SD and remained on study treatment at full dosing for six and eight cycles prior to coming off study; one of these patients had a 20% reduction in cancer antigen 125 (CA125). A patient with refractory metastatic appendiceal carcinoma had SD and continued therapy for six cycles. A male with metastatic colon adenocarcinoma treated at 210 mg has SD and continues full-dose therapy beyond fourteen cycles; he had six prior lines of chemotherapy for metastatic disease, (including flurouracil, oxaliplatin, bevacizumab, irinotecan, panitumumab, anti PD-1 monoclonal antibodies, and an investigational SIRPα antagonist) and has both an ongoing 13% reduction in sum of longest dimensions of the target lesions, and showed a parallel 48% reduction in serum carcinoembryonic antigen (CEA) level. He has remained on zelenirstat for 420 + days, which is a longer duration of treatment and treatment interval than any of the other six prior lines of therapy. No objective responses were yet observed in this study, although radiologic regressions of individual target lesions and minor responses were seen.

**Table 5:** Phase I response data for patients treated with zelenirstat

	Evaluable Patients (*n* = 24)		
Dose level: dose	Best response	No. of patients	Tumor type (No.)
2: 40 mg	SD	1	- Pancreatic adenocarcinoma
5: 140 mg	SD	1	- CRC
6: 210 mg	SD	5	- CRC [2] - Ovarian [2] - Appendiceal carcinoma
7: 280 mg*	SD	1	- Bladder

* Initially dosed at 280 mg daily but after DLT, dose modified to 210 mg daily

*Abbreviations* CRC, colorectal adenocarcinoma NSCLC, non-small cell lung cancer; SD, stable disease

**Fig. 1:**
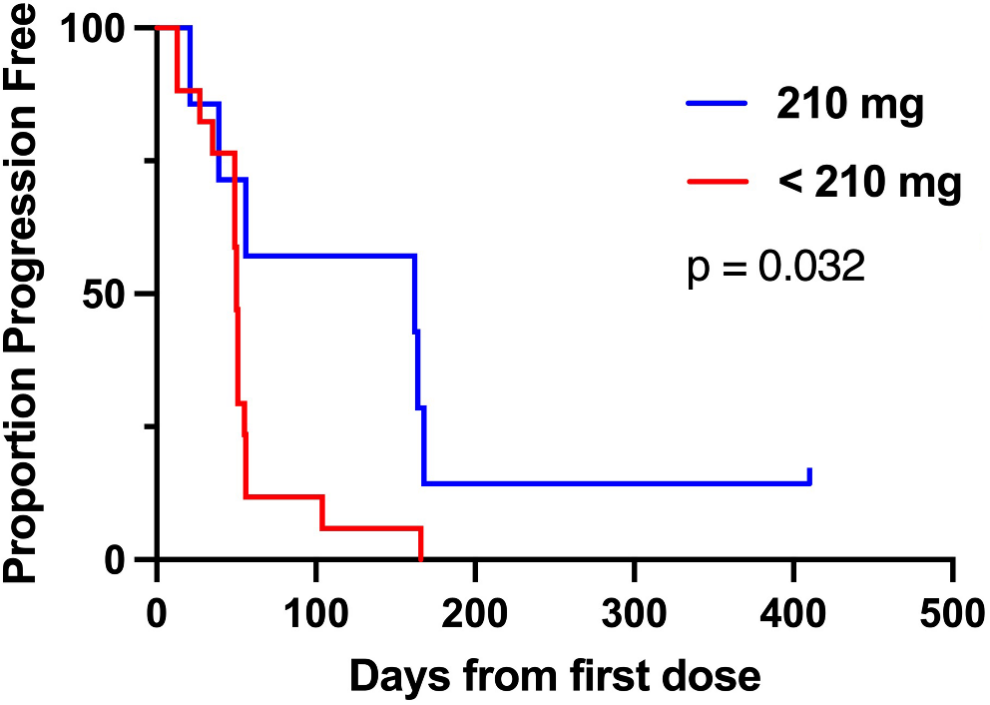
Progression-Free Survival. Kaplan-Meier analysis for time to treatment failure, as measured in days from the first dose of zelenirstat. Patients in the MTD cohort (*n* = 7) of 210 mg daily zelenirstat had statistically significantly longer PFS than patients treated in lower dose cohorts (*n* = 17)

**Fig. 2:**
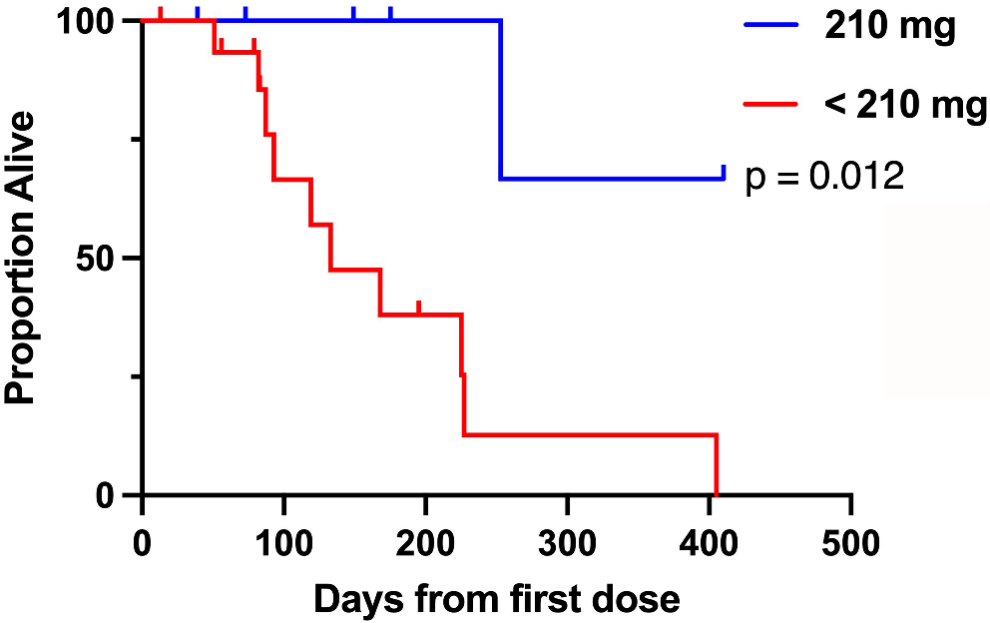
Overall Survival. Kaplan-Meier analysis for overall survival, as measured in days from first dose of zelenirstat. Patients in the MTD cohort (*n* = 7) of 210 mg daily zelenirstat had statistically significantly longer overall survival than patients treated in lower dose cohorts (*n* = 17)

Kaplan-Meier analysis of progression-free survival and overall survival suggests the seven patients treated at 210 mg had significantly better outcomes than those treated with lower doses of zelenirstat **(**Figs. 1 and 2**)**; weighted trajectory analysis confirmed significantly better health status (data on file; [13]).

## Discussion

N-myristoyltransferases are promising new targets for cancer therapy. Because myristoylation is critical to the function of numerous cancer-promoting signaling proteins including Src and SFKs, inhibiting NMTs could dampen survival signals and provide anticancer effects. However, the large number of myristoylated human proteins and the complexity of the affected pathways make it difficult to predict the clinical effects of systemic inhibition of NMTs. Herein, we report the first study of human exposure to any inhibitor of N-myristoyltransferase. Zelenirstat, an orally available, potent pan-NMT inhibitor was given daily to patients with refractory solid tumors and relapsed B-cell NHL. Although dose escalation identified gastrointestinal symptoms as the most frequent adverse events, they were low grade, not dose-limiting up to and including doses of 210 mg daily, and responsive to standard medical management without dose reduction. At doses of 280 mg daily, gastrointestinal toxicities were dose-limiting but reversible. Pharmacokinetic studies confirmed rapid oral absorption, dose-dependent drug exposure, and pharmacokinetic parameters suitable for a once daily administration schedule. Zelenirstat safely achieved blood levels and exposures well above that required for cytotoxicity in cultured human cancer lines.

Anti-cancer activity was a secondary endpoint of this study. Preclinical xenograft models suggested DLBCL was more sensitive to zelenirstat than solid tumor xenografts and given the significant disease burden and heavily pretreated population, we did not anticipate clinical benefit in patients with unselected solid tumours. Nonetheless, eight patients with heavily pretreated advanced solid tumors achieved stable disease as best response **(**Table 5**)**, and four solid tumor patients treated at 210 mg daily appear to have had clinical benefit, receiving prolonged and uninterrupted durations of therapy (two patients who received six cycles, one who received eight cycles, and one other continuing full dose therapy in the fifteenth cycle) with minimal toxicity and some evidence of minor response and reductions in serum tumor markers. The apparent clinical benefit was evident on statistical analysis; progression free survival and overall survival were each significantly better in patients treated with 210 mg compared to those patients treated on lower-dose cohorts **(**Figs. 1 and 2**)**, with the caveat that the treatment allocation was non-randomised and included only a small number of patients.

Apparent clinical benefit was seen in patients with solid tumors including ovarian cancer and colorectal cancer. Ovarian and colorectal cancer was among the zelenirstat-sensitive tumor types in pre-clinical screens and predicted to be sensitive based on transcriptomic analyses [7]. Ovarian cancer metastases have higher NMT1 protein levels than paired ovarian primary cancers, and aggressive ovarian cancers have higher levels of Src, suggesting a possible mechanism for zelenirstat activity in this disease [14]. Src is similarly important for colorectal cancer pathogenesis, as Src activity is required both for epidermal growth factor receptor (EGFR) and vascular endothelial growth factor (VEGFR) signaling, and zelenirstat has potent antiangiogenic effects [15]. Furthermore, zelenirstat inhibits the myristoylation of ADP-ribosylation factor 6 (ARF6) in cultured colon cancer cells, and triggers ARF6 degradation (Pacylex, data on file). ARF6 is required for both the initial plasma membrane localization and recycling of both EGFR [16] and VEGFR [17, 18] back to the plasma membrane after ligand binding; these receptors are otherwise degraded. Furthermore, our previously reported zelenirstat-induced suppression of oxidative phosphorylation [7] has been reproduced in colon cancer cell lines (Pacylex, data on file). The combination of loss of growth factor signaling together with antiangiogenic and metabolic effects may explain the clinical benefit seen in a variety of advanced solid tumors. A phase IIA expansion cohort of this study is now accruing patients with refractory colorectal cancer, and additional studies are planned to better understand the spectrum of zelenirstat activity in solid tumors.

Although the pre-clinical studies of zelenirstat strongly support a potential role in therapy of B-cell NHL, there were relatively few lymphoma patients enrolled in the study, and the four treated patients were in the lower dose cohorts. Given the encouraging safety profile and drug exposure achieved at 210 mg daily greatly exceeds the exposure predicted to achieve anti-lymphoma activity, we have opened a phase IIA expansion cohort of this study and are now accruing patients with R/R B-cell NHL to seek preliminary evidence of activity in this population.

While zelenirstat warrants continued evaluation in phase II monotherapy studies, the drug has a unique mechanism of action and has shown high level preclinical synergy with doxorubicin, cytarabine and venetoclax (Pacylex, data on file), and external beam radiotherapy [19]. Combination therapies, given the low toxicity and ease of administration of zelenirstat, are warranted. These may be particularly appropriate when used with other daily oral therapies or multi-fraction external beam radiotherapy.

Also, on the weight of preclinical evidence for efficacy in AML, the US FDA has granted IND approval, fast-track and orphan drug designations for the development of zelenirstat in relapsed adult AML.

In this first report of any therapeutic human use of N-myristoyltransferase inhibitors, oral zelenirstat achieves therapeutically relevant exposure levels with good safety and tolerability when given on a once daily schedule. The MTD of oral once daily zelenirstat is 210 mg, and gastrointestinal toxicity limits higher doses. Apparent clinical benefit is seen in patients with advanced refractory epithelial cancers, with prolonged stable disease and the absence of cumulative toxicities. These findings strongly support the continued evaluation of oral zelenirstat in advanced cancers, both as monotherapy in selected malignancies, and in combination with other cancer therapies including radiotherapy.

## Data Availability

No datasets were generated or analysed during the current study.
